# Disentangling outbreaks using whole-genome sequencing: concurrent multistate outbreaks of *Salmonella* Kottbus in Germany, 2017

**DOI:** 10.1017/S0950268820000394

**Published:** 2020-02-13

**Authors:** J. Enkelmann, A. von Laer, S. Simon, A. Fruth, R. Lachmann, K. Michaelis, M. Borowiak, S. Gillesberg Lassen, C. Frank

**Affiliations:** 1 Department of Infectious Disease Epidemiology, Robert Koch Institute, Berlin, Germany; 2 Postgraduate Training for Applied Epidemiology, Robert Koch Institute, Berlin, Germany; 3 European Programme for Intervention Epidemiology Training, ECDC, Sweden; 4 Department of Infectious Diseases, Robert Koch Institute, Wernigerode, Germany; 5 Department Biological Safety, German Federal Institute for Risk Assessment (BfR), Berlin, Germany; 6 Department for Infectious Disease Epidemiology and Prevention, Statens Serum Institut, Copenhagen, Denmark

**Keywords:** Food-borne infections, Germany, molecular epidemiology, outbreaks, salmonellosis

## Abstract

In June 2017, an outbreak of S*almonella* Kottbus infection was suspected in Germany. We investigated the outbreak with whole-genome sequencing (WGS) and a case–control study. Forty-six isolates from 69 cases were subtyped. Three WGS clusters were identified: cluster 1 (*n* = 36), cluster 2 (*n* = 5) and cluster 3 (*n* = 3). Compared to controls, cluster 1 cases more frequently consumed raw smoked ham (odds ratio (OR) 10, 95% confidence interval (CI) 1.2–88) bought at supermarket chain *X* (OR 36, 95% CI 4–356; 9/10 consumed ham *Y*). All four cluster 2 cases interviewed had consumed quail eggs. Timely WGS was invaluable in distinguishing concurrent outbreaks of a rare *Salmonella* serotype.

## Background

In Germany, salmonellosis is a notifiable disease. *Salmonella enterica* subsp. *enterica* serotype Kottbus (*S*. Kottbus) is a rare serotype with three to four notifications per month [1]. *S.* Kottbus has been repeatedly isolated from poultry and poultry meat and has been found in cattle, pork, pigs and reptiles [2]. Bottled water, sprouts and human milk have been identified as vehicles in previous *S.* Kottbus outbreaks [3–5].

### The event

In July 2017, the notifications of *S.* Kottbus infections increased, particularly in the coastal area of Schleswig-Holstein, Germany. We conducted an outbreak investigation to identify the source of the outbreak and to implement control measures.

## Methods

### Case definition and case finding

Outbreak cases were defined as individuals living in Germany with *S.* Kottbus infection, symptom onset between 20 June and 31 August 2017 and no travel abroad in the week before symptom onset. If the date of onset was not available, the date of sample receipt at the National Reference Centre for *Salmonella* and other Bacterial Enterics (NRC) between 26 June and 31 August 2017 was used.

A *S.* Kottbus case was defined as confirmed if subtyping, either using pulsed-field gel electrophoresis (PFGE) or whole-genome sequencing (WGS), showed that it belonged to an outbreak cluster. A *S.* Kottbus case without subtyping, but with an epidemiological link to a confirmed case, was defined as probable.

### Microbiological investigations

*S*. Kottbus isolates from cases voluntarily sent to the NRC by diagnostic laboratories were subtyped using PFGE following *XbaI* macrorestriction according to the PulseNet protocol and/or WGS [6].

DNA was isolated with the GenElute Bacterial Genomic DNA Kit (Sigma-Aldrich Chemie GmbH, Munich, Germany) according to the manufacturer's instructions and used to generate libraries by using the Nextera XT DNA Library Preparation Kit from Illumina. Genomic sequences were obtained by short-read sequencing using an Illumina MiSeq benchtop sequencer performed in paired-end mode using a v3 chemistry-based cartridge 600 (600-Cycle Reagent Kit, Illumina).

The multi-locus sequence type (MLST) based on seven housekeeping gene loci was extracted from whole-genome data [7]. The bioinformatics analysis for cluster detection was performed with an *ad hoc* cgMLST-scheme for *S. enterica* based on 2143 loci using Ridom SeqSphere^+^ [8]. Raw reads from one exemplary isolate belonging to the outbreak cluster were uploaded to the European Centre for Disease Prevention and Control (ECDC) server to allow comparison with isolates from other European countries. Sequences, provided from Public Health Institutes of other countries, were included in the gene-by-gene analysis.

### Epidemiological investigations

#### Case–control study

We telephone-interviewed adult *S.* Kottbus cases residing or exposed in the states Schleswig-Holstein, Hamburg and Mecklenburg-Western Pomerania (hereafter ‘Northern Germany’) with symptom onset between 20 June and 16 August 2017. In households with more than one case, only the patient with the earliest symptom onset was included.

We recruited four frequency-matched controls per case (by age group (25–49, 50–64, 65–85 years), sex and district of residence). Controls were identified by a research service institute using random digit dialling of landline numbers. If there was a suitable control person in the household, they were telephone-interviewed.

Using a structured questionnaire, participants were asked about symptoms, food products consumed, and where they were consumed up to 1 week prior to illness/interview and up to 2 weeks for supermarkets.

Only a subset of controls were asked about ham consumption, as it evolved as the suspected vehicle after the interviews of controls had already started and call-backs were not possible. If ham was consumed, pictures were provided to cases to identify specific products.

In the analysis, we excluded patients with subtyping results not consistent with cluster 1 and included patients exploratively interviewed, as the final hypothesis had not been formulated based on these interviews; for these cases, no controls were recruited. To adjust for the matching variables, we grouped cases and controls by age group (25–55, 56–85 years), sex and state of exposure. We conducted univariable and multivariable analysis and estimated adjusted odds ratios (aOR) and their 95% confidence intervals (CI) using conditional logistic regression with STATA version 14.1 (StataCorp, College Station, Texas, USA). In the analysis, uncertain exposure was considered as missing. Exposures with the smallest *P*-values in the univariable analysis (⩽0.08) and possibly originating from the same food source as the implicated vehicle were included in the multivariable model. To test for the independence of variables, we performed *χ*^2^ tests. Regiograph Analyse (GfK SE, Nuernberg, Germany) was used to create maps.

#### Interviews of cases not included in the case–control study

A short questionnaire including two relevant exposures identified during the case–control study was distributed to local public health offices for interviews with cases not included in the case–control study.

#### Food and environmental investigations

The German Federal Institute for Risk Assessment provided *S*. Kottbus sequences from routine non-human microbiological surveillance in 2017. Food samples taken during the outbreak investigation and retention samples of the implicated product were microbiologically tested.

#### International inquiry

On 22 August 2017, we informed other countries about the increase in *S*. Kottbus cases via the ECDC's Epidemic Intelligence Information System (EPIS) [9]. Countries were asked to report the increases of *S*. Kottbus notifications or *S.* Kottbus patients with travel to Germany and to provide a sequence to compare with the sequences of German clusters.

## Results

We identified 69 cases in 13 states in Germany. Median age was 55 years (range 0–91) and 55% were female (*n* = 38).

### Microbiological investigations

Forty-six isolates from 69 cases were subtyped using PFGE (*n* = 30) and/or WGS (*n* = 39). PFGE and WGS were concordant in their identification of cluster 1 (*n* = 34 cases). Another PFGE cluster was subdivided by WGS into cluster 2 (*n* = 5) and cluster 3 (*n* = 3) (Table 1). Four cases had isolates with PFGE patterns compatible with more than one cluster (*n* = 2) or did not match any of the identified clusters (*n* = 2).

**Table 1.** Subtyping results of human *S.* Kottbus isolates (*n* = 46), Germany, June–August 2017

Cluster	Correlating PFGE-pattern^a^	Number of isolates subtyped			
		Total	Only WGS	Only PFGE	WGS + PFGE
1	13, 13a, 14, 5var3.1	34	13	4	17
2	5var1	5	1	0	4
3	5var1	3	2	0	1
Not cluster 1, 2 or 3	5var4	1	0	0	1
	15	1	0	1	0
Cluster unknown	13b	1	0	1	0
	5var1	1	0	1	0

a Internal nomenclature.

**Fig. 1.**
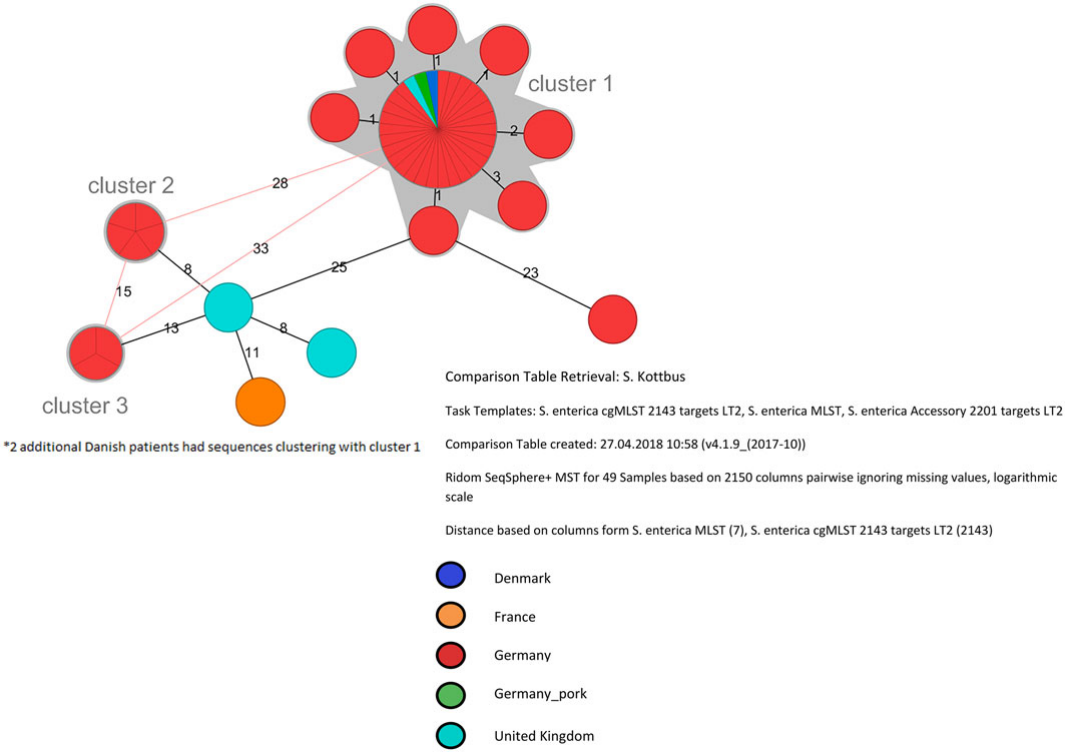
cgMLST-based Minimum Spanning Tree of *S*. Kottbus isolates, June–August 2017.

Within the three WGS clusters, the maximum pairwise distance was four loci, while different clusters were 15–33 loci apart (Fig. 1). All three clusters belonged to 7-locus sequence type ST212. Denmark, Ireland, the UK and France provided *S*. Kottbus sequences for comparison. Two sequences, one from Denmark and one from the UK, were indistinguishable from cluster 1.

### Epidemiological investigations

#### Description of *S*. Kottbus cases by cluster

**Fig. 2.**
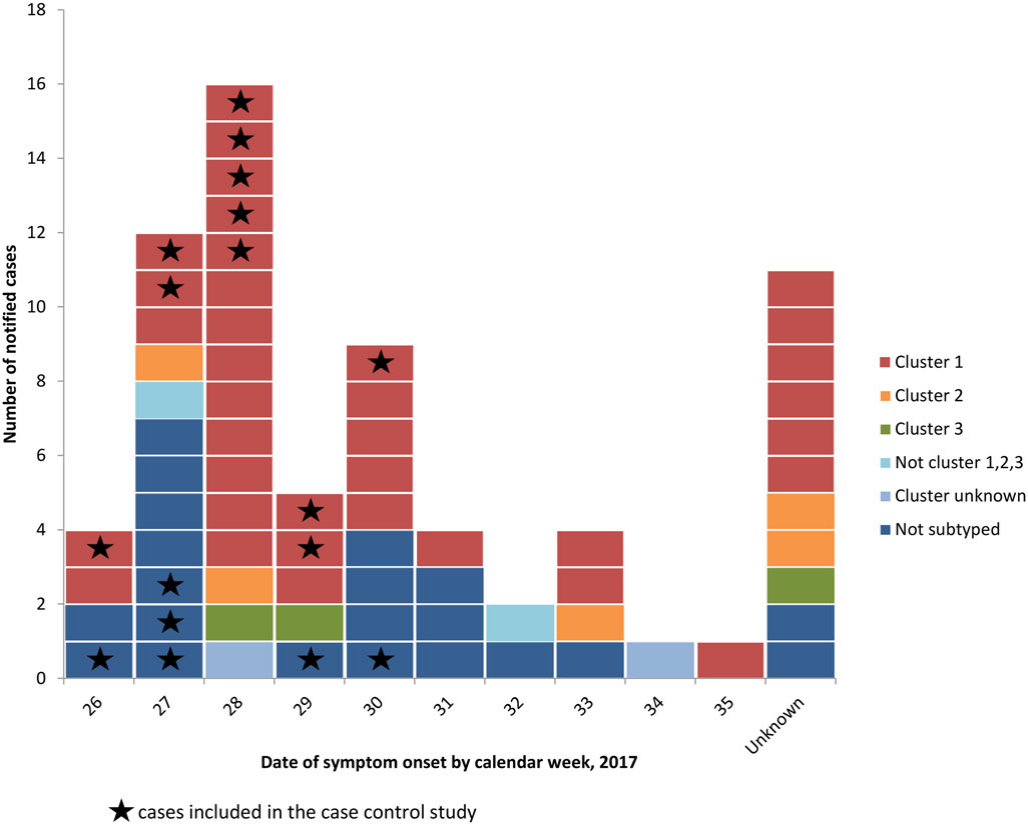
Epidemic curve of *S*. Kottbus cases by date of symptom onset and microbiological cluster (*n* = 69), Germany, June–August 2017.

**Fig. 3.**
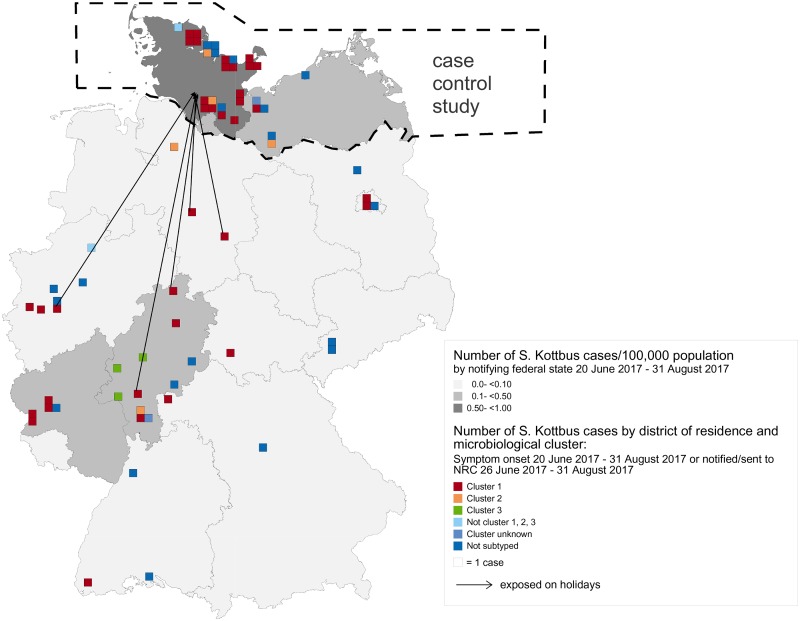
Incidence and geographical distribution of *S*. Kottbus cases according to cluster (*n* = 69), Germany, June–August 2017.

Of 36 cluster 1 cases, 34 were confirmed and two probable, and 23 (64%) were exposed in Northern Germany, half were female and the median age was 54 years (range 2–91) (Figs. 2 and 3).

Five confirmed cluster 2 cases (80% female, median age 58 years) were exposed in five states.

All three confirmed cluster 3 cases (100% male, range 31–71 years) were residing in Hesse.

#### Case–control study

Ninety-six controls and 17 cases (11 confirmed and six probable cluster 1) were included in the case–control study.

Of 14 cases reporting raw smoked ham (hereafter ‘ham’) consumption, 10 (71%) had eaten ham from supermarket chain *X*. Of those, nine cases had eaten a specific ham product *Y* and one a slightly different product with a similar package. Four cases (29%) reported the consumption of any type of raw minced meat, including two of the three cases with no or uncertain ham consumption.

Consumption of ham from supermarket chain *X* and raw minced meat had the highest point estimates (OR 37, 95% CI 3.7–356 and OR 4, 95% CI 0.9–18.3, respectively, Table 2). As the exposures were independent and raw minced meat could explain two additional cases, both were included in the multivariable model.

**Table 2.** Univariable and multivariable analysis of exposures of cases and controls, case–control study, *S.* Kottbus outbreak, Northern Germany, June–August 2017

Exposure	Cases (*n* = 17)^a^		Controls (*n* = 96)^a^		Univariable^a^		Multivariable^a^	
	*n*/*N*	%	*n*/*N*	%	aOR^b^	95% CI	aOR^b^	95% CI
Raw smoked ham from supermarket chain *X*	10/14	71	3/34	9	36.5*	3.7–355.6	39.4*	3.0–510.8
Raw smoked ham	14/15	93	16/34	47	10.4*	1.2–88.4	–	–
Specific ham *Y* from supermarket chain *X*	9/10	90	Not asked	–	–	–	–	–
Raw minced meat	4/14	29	11/96	11	4.0	0.9–18.3	4.7	0.3–64.4
Tea sausage spread	3/11	27	3/34	9	3.4	0.5–21.8	–	–
Turkey	5/13	38	16/96	17	2.8	0.8–11.0	–	–
Chicken	6/11	55	43/96	45	1.6	0.4–6.8	–	–
Kebab	3/16	19	13/96	14	1.5	0.3–6.5	–	–
Contact to seagulls	2/16	13	9/96	9	1.3	0.3–6.9	–	–
Fish	10/17	59	49/96	51	1.2	0.4–3.5	–	–
Raw or soft-boiled egg	7/14	50	41/96	43	1.2	0.3–3.9	–	–
Unheated sprouts	1/14	7	6/95	6	1.2	0.1–11.2	–	–
Potato salad	4/15	27	23/96	24	1.1	0.3–3.6	–	–
Ice cream	5/16	31	44/96	46	0.5	0.2–1.8	–	–
Raw vegetable salad	8/12	67	28/34	82	0.3	0.6–1.6	–	–

a Uncertain consumption was coded as missing.

b Adjusted for age group, sex and state of exposure.

*P-value <0.05.

Multivariable analyses showed similar point estimates (Table 2). Cases had a 39 times higher odds to have consumed ham from supermarket chain *X* (OR 39, 95% CI 3–511) and a 4.7 times higher odds to have consumed raw minced meat (OR 4.7, 95% CI 0.3–64.4) than controls. Only ham consumption was statistically significant.

#### Interviews of cases not included in the case–control study

Twenty cases not included in the case–control study were interviewed with a short questionnaire inquiring about the consumption of ham and quail eggs. The hypothesis of quail eggs being the vehicle for cluster 2 evolved when two cases initially interviewed for the case–control study reported this unusual exposure and subsequent subtyping revealed they both belonged to cluster 2, resulting in their exclusion from the case–control study.

Of the eight interviewed cluster 1 cases outside Northern Germany, four had consumed ham; three had consumed ham *Y* or a similar ham product from a different supermarket chain. Of the five probable cluster 1 cases, three had eaten or handled ham.

All three interviewed cluster 2 cases and one case belonging to either cluster 2 or 3 recalled handling and/or consuming quail eggs (Table 3). Two cases reported eating the eggs raw or soft-boiled. The quail eggs were bought at two different supermarket chains. A common origin could not be excluded. Cases stated consuming quail eggs for health reasons, as a nourishing food or because of chicken egg allergies. No other case reported this exposure.

**Table 3.** Ham and quail egg consumption by *S*. Kottbus cases not included in the case–control study by subtyping results (*n* = 20), Germany

Exposure	Cluster 1^a^ (*n* = 8)		Cluster 2^a^ (*n* = 3)		PFGE: Cluster 2/3^a^,^b^ (*n* = 1)		Cluster 3^a^ (*n* = 2)		No subtyping results^a^ (*n* = 5)		No cluster^a^ (*n* = 1)		Total^a^ (*n* = 20)	
	*n*/*N*		*n*/*N*		*n*/*N*		*n*/*N*		*n*/*N*		*n*/*N*		*n*/*N*	
Quail egg	0/7		3/3		1/1		0/2		0/4		0/0		4/17	
Raw smoked ham	4/7		0/3		0/1		1/1		3/5		1/1		9/16	

a Uncertain consumption was coded as missing.

b PFGE: 5var1: compatible with cluster 2 or cluster 3, no WGS available.

No common food item was identified for two interviewed cluster 3 cases.

#### Food and environmental investigations

A *S.* Kottbus isolate from pork intended for animal feed from April 2017 was indistinguishable from cluster 1 by WGS. A link to ham production could not be established.

The ham producer presented negative microbiological test results from the company's internal control conducted at the beginning of August 2017. No leftover ham products from affected households were available for analysis. All tested samples of ham and quail eggs collected from supermarkets late in the outbreak investigation were negative for *Salmonella* spp.

#### International inquiry

Fourteen countries replied on EPIS. Based on WGS, five patients belonging to cluster 1 were reported. One patient from the UK (symptom onset in May 2017) without travel history had handled and consumed raw pork; details on the type were not provided. Of the four patients from Denmark (symptom onset April–September 2017), three were interviewed and reported buying food in German supermarkets in Denmark. Two patients had travelled to Germany in the week before symptom onset; one recalled consuming ham *Y* bought in Northern Germany from supermarket chain *X*.

## Discussion

WGS of *S.* Kottbus isolates revealed three co-circulating clusters in Germany between June and August 2017.

Thirty-six cases belonged to the largest outbreak cluster (cluster 1). Cluster 1 patients were also identified in Denmark and the UK. In Germany, this strain had not previously been detected in humans.

Of all interviewed confirmed cluster 1 cases, 82% could be explained by raw smoked ham. Ham *Y* from supermarket chain *X* was identified by nine out of 10 cases asked. No other food item could explain as many cases. In addition, one patient from Denmark who had been in Northern Germany at the time of exposure confirmed eating raw smoked ham *Y* from supermarket chain *X*.

The matching isolate from pork intended for animal feed shortly before the outbreak further supports ham as the likely vehicle, even if a link between the isolate and ham *Y* could not be established. *S.* Kottbus could not be detected in food samples, but they were collected late during the outbreak investigation. By September 2017, the number of *S*. Kottbus notifications fell to the expected number.

Raw smoked ham and raw minced pork have been linked repeatedly to outbreaks with other *Salmonella* serotypes [10–15] and *S*. Kottbus has previously been isolated from pigs and pork in Germany [2]. It therefore seems possible that other pork products, especially if consumed raw, may have contributed to this outbreak. Two of the three cases with no or uncertain ham consumption in our case–control study had eaten raw minced meat, potentially containing pork. Although consumption of raw minced meat could possibly explain additional cases and was more commonly reported by cases than controls in our case–control study, this result was not statistically significant and the exposure ‘only’ explained 29% of cases. As both products originate from the same food source, it remains unclear if raw minced meat additionally contributed to the cluster 1 outbreak.

Quail eggs were the likely source for the smaller second identified cluster (cluster 2). *S.* Kottbus has previously been detected in quails [2]. All interviewed cluster 2 cases reported the consumption of quail eggs. This seems disproportionately high, especially as only 1% of German adults participating in a representative food survey in 2017 reported quail egg consumption within the last week (unpublished results, personal communication B. Rosner, RKI). Quail eggs should be considered as a potential vehicle in *Salmonella* outbreaks.

Although *S*. Kottbus is a rare *Salmonella* serotype, molecular subtyping, especially WGS, was essential in distinguishing between different clusters and in identifying concurrent *S*. Kottbus outbreaks. As WGS was not conducted routinely in Germany before 2017, we do not know if clusters 2 and 3 might have previously contributed to the background rate of *S*. Kottbus infections.

Without WGS, misclassification of cases would have weakened the measures of association and impeded the identification of the vehicle of the cluster 1 outbreak and obscured the strong common exposure of quail egg consumption reported by cluster 2 cases.

## Conclusion

Our investigation highlights the importance of subtyping. WGS especially was essential in identifying the concurrent outbreaks of a rare *Salmonella* serotype and supported the epidemiological investigation by minimising case-misclassification. WGS was therefore valuable in identifying raw smoked ham and quail eggs as likely vehicles in concurrent *S*. Kottbus outbreaks.
